# Why does the number of dangerous species of scorpions increase? The particular case of the genus *Leiurus* Ehrenberg (Buthidae) in Africa

**DOI:** 10.1590/1678-9199-JVATITD-2020-0041

**Published:** 2020-06-17

**Authors:** Wilson R. Lourenço

**Affiliations:** 1 Muséum national d’Histoire naturelle, Sorbonne Universités, Institut de Systématique, Evolution, Biodiversité (ISYEB), UMR7205-CNRS, MNHN, UPMC, EPHE, CP 53, 57 rue Cuvier, 75005 Paris, France.

**Keywords:** Scorpion, Systematics, New noxious species, Africa, Leiurus

## Abstract

The aim of this contribution is to bring some precise information on the reasons why the number of noxious scorpion species is constantly growing. This fact is directly associated with the zoological research on the domains generally defined as systematics and taxonomy. The classification of any zoological group is in most cases a source of problem for most biologists not directly involved with this almost confidential aspect of the zoological research. Much information has been gathered and published over two centuries on the classification but it is remains poorly accessible and too technical for non-experts. The exposed example could be taken from several groups of scorpions possessing infamous species, but the choice went to the genus *Leiurus* Ehrenberg, 1828 distributed from North Africa to the Middle East. Maybe this contribution will help to explain why so numerous cases of species misidentification are regularly present in the general literature devoted to scorpion venoms and incidents.

## Background

In recent years, in a series of publications addressed to the readers of the *Journal of Venomous Animals and Toxins including Tropical Diseases*, I attempted to bring general information on scorpions and scorpionism, using the best possible didactic approach, in order to be understandable to non-specialists whose research embraces scorpions in several fields such as venom toxins and public health [1,2,3,4,5,6,7]. Most of the information previously supplied concerned historical aspects of scorpion studies, but also several questions around their taxonomy, evolution, geographic distribution and life history strategies [1,2,3,4,5,6,7].

The classification of any zoological group (botanical classification is a distinct matter) is in general a source of problems for most biologists not directly involved with this quite particular and almost confidential aspect of the zoological research. Abundant information has been published for more than two centuries on the classification (generally defined as systematics or taxonomy) of scorpions, but it is almost exclusively available in highly specialized literature, normally too technic for non-experts and worse, so scattered that it becomes unavailable for non-experts on the subject. This subject is normally ruled by the International Code of Zoological Nomenclature, but mainly because of a ‘bad tradition’ systematics can be treated also by non-academic people creating therefore an enormous amount of very poor and even totally erroneous decisions.

The number of noxious scorpion species globally cited in the literature remained stable for several decades and in general limited to 20 to 25. Only more recently, a higher number of about 50 dangerous species was recorded in some publications (see Lourenço [3] for details). Consequently, one question is often addressed: Why does the number of dangerous species of scorpions increase? The answer is quite simple. Since research on systematics and taxonomy is always progressing more and more new species of scorpions are discovered and described, that is, named. These new nominations necessarily improve the total number of species. Naturally not all species described are noxious; quite many are totally harmless for humans and nobody will even pay attention to their increasing numbers. Nevertheless, within the group containing dangerous species many novelties are also discovered and named.

The example presented in this text is one among the several hundred I was able to treat on my personal research on scorpion classification (systematics and taxonomy) performed during more than 45 years. All the studies on the systematics and taxonomy need, however, to be considered as largely incomplete, since our global knowledge on scorpions’ classification presents always numerous gaps and unfortunately also quite many very weak or even incorrect contributions. Nevertheless, the proposition of one particular example seems to be useful to illustrate why the scorpion nomenclature constantly changes, what can be a source of confusion or misunderstanding for the non-expert readers of the journal. I can only expect that this presentation will be welcome to a large audience.

## **One particular example: the genus *Leiurus* in Africa**

Among the different generic groups of scorpions containing noxious species to humans, examples could be taken from several such as *Androctonus* Ehrenberg, *Buthus* Leach, *Centruroides* Marx or *Tityus* C. L. Koch which show increasing numbers of new species [3,4]. Nevertheless, I decided to select one example from the genus *Leiurus*, which certainly contains some of the most infamous species of scorpions, starting with its original species *Leiurus quinquestriatus* (Ehrenberg, 1828). 

**Figure 1.**
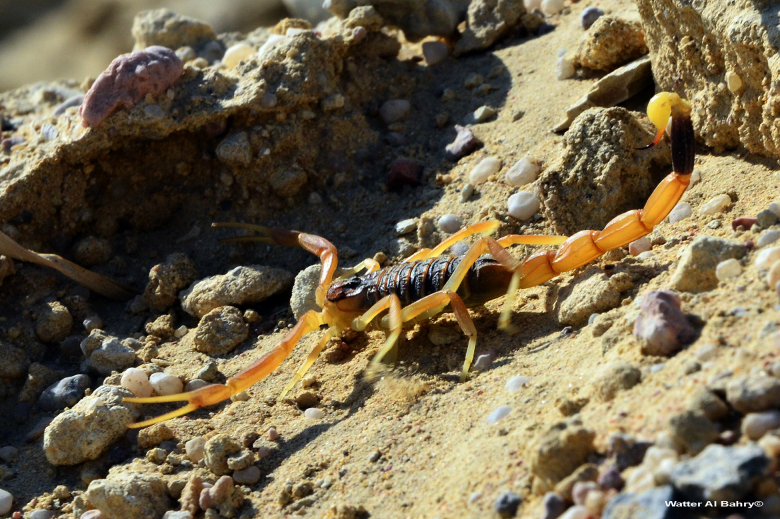
Pre-adult male of *Leiurus quinquestriatus* in its natural habitat, Hurghada Eastern Desert, Red Sea, Egypt (copyright by W. Al Bahry, reproduced with permission).

**Figure 2.**
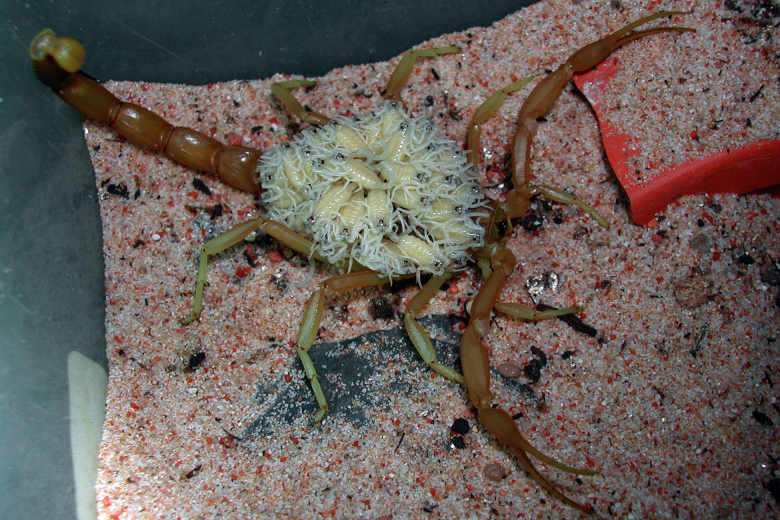
Female of *Leiurus quinquestriatus* in captivity carrying first-instar juveniles. According to Thornton [33] brood size can globally vary from 40 to 60 (copyright by E. Ythier, reproduced with permission).

The genus *Leiurus* Ehrenberg, 1828 was represented over many decades by a single species, *Leiurus quinquestriatus*, containing two subspecies, *L. quinquestriatus quinquestriatus* (Ehrenberg, 1828) and *L. quinquestriatus hebraeus* (Birula, 1908) [8, 9,10,11,12]. *Leiurus quinquestriatus* seems to be a common species in certain regions of Egypt (Figures 1 and 2), Sinai and Sudan. Nevertheless, the precise identity of some regional populations from these areas requires yet further investigation [13]. Contrarily, *L. hebraeus* Birula, 1908 (now recognized as a valid species) is largely distributed in Israel and nearby countries [14,15,16]. *Leiurus* species are among the most common scorpions of desert faunas and, in particular in certain regions of Sudan, and especially around Khartoum and Omdurman, but also in Egypt and Sinai. 

*Leiurus* species secrete one of the most harmful venoms among buthid scorpions in general, and are responsible for severe human incidents. Fortunately, the amount of venom produced by an average sting is rather small (0.225 mg) and, consequently, the lives of adult humans are seldom endangered, although the Sudanese population of *Leiurus* is a significant cause of death among small children [17]. Even if the incidents caused by *Leiurus* species may be considered as rather severe, these are much less frequent, for instance, than those caused by species of *Androctonus*. This is certainly due to the fact that human populations are much less dense in the regions where *Leiurus* species are distributed. Because of their infamous reputation as very dangerous scorpions, the toxins of both *L. quinquestriatus* and *L. hebraeus* have been the subject of numerous biochemical studies [18,19].

Many, if not most, aspects of the taxonomy of the genus *Leiurus* remained confused for many decades. A historical account of the taxonomy of the genus *Leiurus* shows that when the original description of *Androctonus* (*Leiurus*) *quinquestriatus* Ehrenberg, 1828 [20] took place, this species was originally placed in the genus *Androctonus* and *Leiurus* was considered only a subgenus of *Androctonus*. This demonstrates the stage of indecision in the classification on the early 19^th^ century. Several subsequent authors such as Kraepelin in 1891 [21] registered *Leiurus* as a synonym of the genus *Buthus* Leach. Finally, Vachon in 1949 [22] established *Leiurus* as a separate genus with only one species *Leiurus quinquestriatus*. Vachon [22] was confident that this genus was monotypic, but refrained from revising its intraspecific structure. Two subspecies were considered to be valid by Vachon [22]: *Leiurus quinquestriatus quinquestriatus* (Ehrenberg, 1828) and *Leiurus quinquestriatus hebraeus* (Birula, 1908). Subsequently, the systematic position of *Leiurus quinquestriatus hebraeus* (now recognized as a valid species) has been reviewed by Levy et al. [16], who presented tables that differentiate this subspecies from *L. q. quinquestriatus*. The position of the two subspecies was considered again by Levy and Amitai [23].

Only in recent years, totally new species were finally described for the genus *Leiurus*. The description which really changed most conservative views about this group of scorpions was that of *Leiurus jordanensis* Lourenço, Modry & Amr, 2002 from Jordan [8]. Just a few years later, *Leiurus savanicola* Lourenço, Qi & Cloudsley-Thompson, 2006 [9] from Cameroon was described, representing the second confirmed species from Africa.

In a study on Middle Eastern *Leiurus*, Lowe et al. [13] proposed, in a rather extensive article, a full revision of the genus *Leiurus*, but dealing mainly with the populations from the Arabian Peninsula. The status of some old species was revalidated, one recently described species was placed in synonymy, one subspecies was raised to species and four new species were described. This elevated the total number of species in the genus *Leiurus* to ten. The characters used by these authors to define the species, as well as the proposed dichotomic key are however, rather difficult to be used. Nevertheless, it is possible to agree with these authors and, in particular with their opinion about the African species, stated as follows:

Our findings show that, like many other scorpion genera, *Leiurus* is comprised of an assemblage of allopatric or parapatric species spread across different regions separated by physiographic barriers, each adapted to local environments and substrates. Additional species diversity may emerge when other local populations are analyzed in more detail, for example those in southern Sinai and in more central parts of North Africa [13]*.*

Obviously, the status of the African populations of *Leiurus* was largely neglected and is still the subject of new studies. An initial approach in this direction leaded to the description of several new species, namely *Leiurus somalicus* Lourenço & Rossi, 2016 from Somalia, *Leiurus hoggarensis* Lourenço, Kourim & Sadine, 2018 from the South of Algeria and *Leiurus ater* Lourenço, 2019 from the Tibesti Mountains in Chad [10,11,12]

Other populations or citations for Africa remain enigmatic or ambiguous. In his monograph on the scorpions of North Africa, Vachon [24] also referred to several specimens collected in Fezzan (Libya) as *L. quinquestriatus*. However, it is quite possible that this population do corresponds to *Buthus quinquestriatus libycus* Birula, 1908 (= *Leiurus quinquestriatus libycus*). Nevertheless, only the study of more fresh material from Libya will allow a confirmation of this suggestion. Duval et al. [25] referred to one specimen of *Leiurus quinquestriatus* collected in the northwest section of the Algerian desert, a zone located outside of the known range of distribution of the species. The studied specimen is considered lost so only new collected material in the area will allow a clarification. In a generalist study, Goyffon & Billiald [26] suggested the presence of *L. quinquestriatus* in Mauritania; however, in absence of any evidence. In a similar study Goyffon et al. [27] associated *L. quinquestriatus* to a population in Mali. This identification proceeded by non-experts is most certainly erroneous and this Malian population probably has more connections with *L. hoggarensis* or even with an undescribed form. 

In the present contribution the example I will use to illustrate the description of a new species will be based on material collected precisely in Mauritania, in a region of the Sahel. Until now, most known species of *Leiurus* are typical of desert formations, with one exception being that of *Leiurus savanicola* which was collected is the transitional zone between the Sahel and savannah formations, in a burrow under a rock, consequently out of the typical desert formations where *Leiurus* species are commonly found.

**Figure 3.**
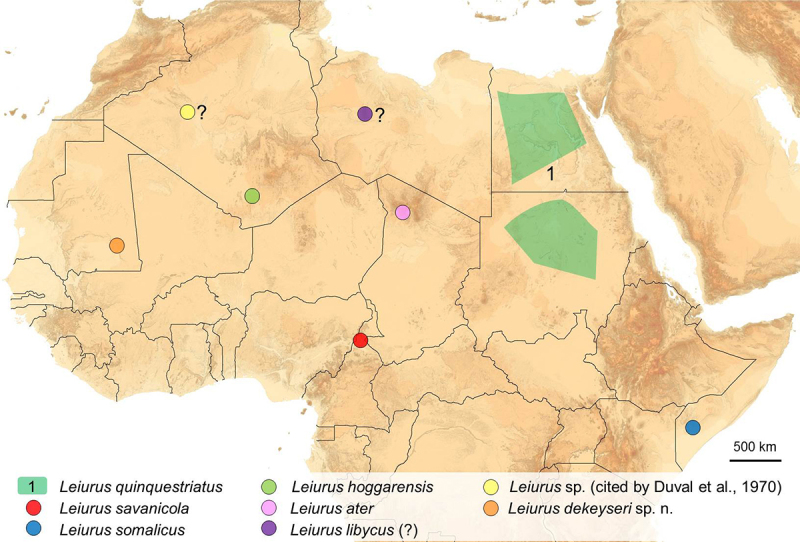
Map of the north portion of Africa showing the distribution of the known *Leiurus* species.

The new geographical distribution of the genus *Leiurus* can be summarized as follows: Algeria, Chad, Egypt, Ethiopia, Libya, Mali, Mauritania, Niger, Somalia, Sudan, and Tunisia in Africa; and Sinai, ?Iraq, Israel, Jordan, Kuwait, Lebanon, Oman, Qatar, Saudi Arabia, Syria, Turkey, United Arab Emirates and Yemen in Asia. The major population in Africa clearly corresponds to the species *L. quinquestriatus* with the other species located in more patchy distributions (Figure 3). The Isthmus of Suez apparently corresponds to the border between the African species of *Leiurus* and those distributed in the Middle East [16,23]. The species *Leiurus savanicola* only known from the Sahel in Cameroon and *Leiurus somalicus* only known from the South of Somalia represents the most Southern records on the distribution of the entire genus *Leiurus*.

## Methods

Even in taxonomic studies the presentation of methods is important in order to facilitate the reproduction of several parameters related with the results. Naturally, these are globally less detailed than those used in experimental biology, but some topics have a major importance.

Authors can indicate the used equipment such as the Wild M5 stereo-microscope with a drawing tube and ocular micrometer used in the present study for drawings and measurements; measurements normally follow a previous reference such as Stahnke [28]. Nomenclature is also important and has to be based on previous references. For trichobothrial notations Vachon [29] is used and for the general morphological terminology Hjelle [30] is a good reference. One major aspect in any taxonomic study is to precisely indicate the original locality where the new species was collected. This point is indicated together with the list of the material used for the description. The choice of a name for the new taxon (new species) is totally free. The author(s) can name the new species after the region where it was collected, can honor its collector or base the new name on any morphological characteristic of the new species. The material used for the description becomes a referential material called type material. The first element used is defined as the holotype and the following specimens, if existent, are defined as paratypes. All the type material should *a priori* be deposited exclusively in official academic institutions; however, many non-academic people involved in taxonomy often do not follow this rule and keep the type material in ‘uncontrolled private collections’.

The description requires three essential aspects: a diagnosis in which the major characteristics are listed, the description itself where authors are free to use their personal style and a table of relationships with the most closed related species already known. This presentation is normally closed with the standard morphometric values of the types. 

## Taxonomic treatment

Family Buthidae C.L. Koch, 1837

Genus *Leiurus* Ehrenberg, 1828

**Figure 4.**
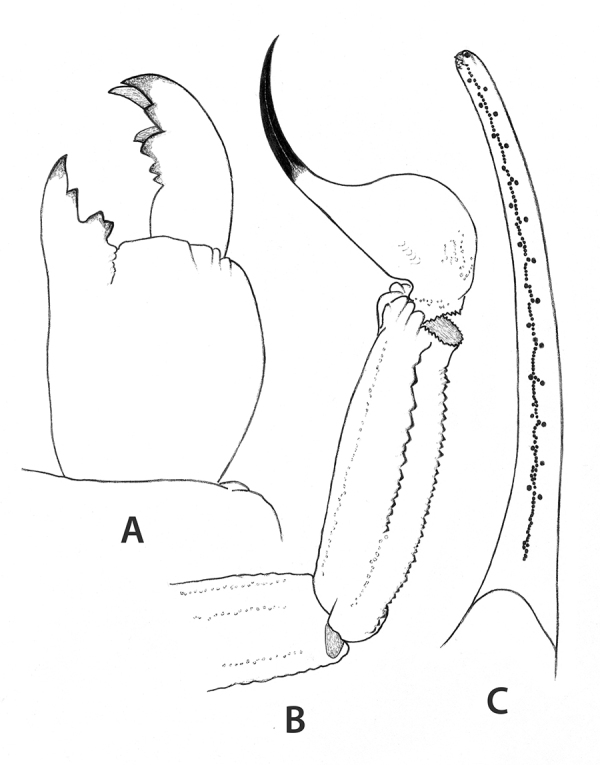
*Leiurus dekeyseri* sp. n. Female holotype. **(A)** Chelicera, dorsal aspect. **(B)** Metasomal segment V and telson, lateral aspect. **(C)** Cutting edge of movable finger, showing rows of granules.

**Figure 5.**
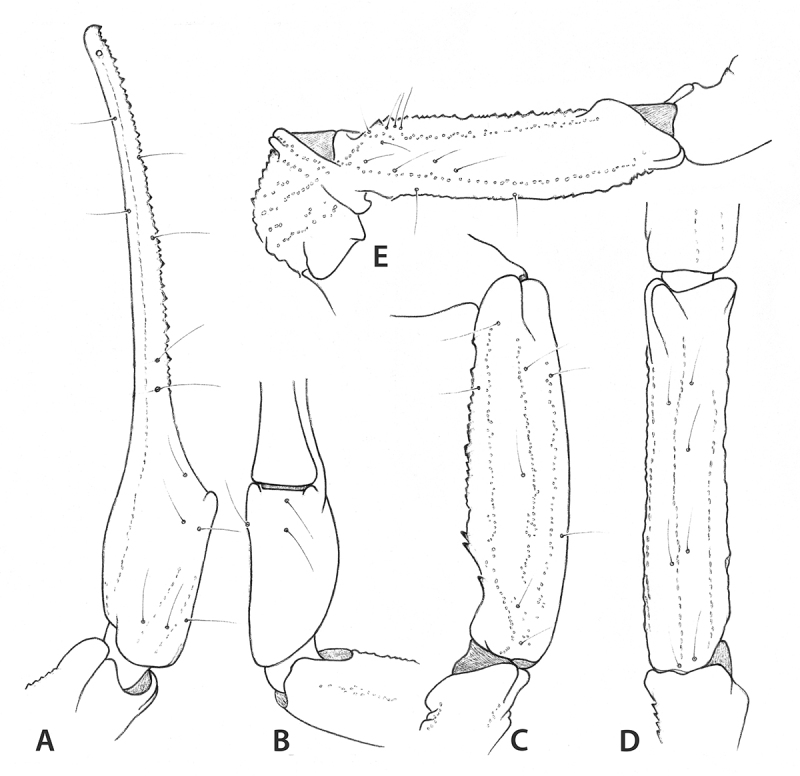
*Leiurus dekeyseri* sp. n. Female holotype. Trichobothrial pattern. **(A, B)** Chela, dorso-external and ventral aspects. **(C, D)** Patella, dorsal and external aspects. **(E)** Femur, dorsal aspect.

*Leiurus dekeyseri* sp. n. (Figures 4 and 5)

Type material: Mauritania (AOF), between Néma and Bassikounou, X/1962 (P. L. Dekeyser). The holotype will be deposited in the Museu Nacional, Rio de Janeiro, RJ, Brazil, as a contribution to the reposition of the collections destroyed by fire in 2018.

Patronym: specific name honors Pierre Louis Dekeyser, my first zoological mentor, who also collected the holotype during one of his multiples field trips in Occidental Africa.

Diagnosis: Scorpion of moderate size when compared with the other species of the genus, having a maximum total length of 71.5 mm for female. The ground color is pale yellow to almost whitish for both the body and appendages. Only the ventral aspect of metasomal segment V is slightly infuscate; other metasomal segments are pale yellow. Ocular tubercle strongly prominent. Pectines with 28-27 teeth. Median carinae on sternites III-IV moderately to strongly marked; sternite VII with mediate intercarinal surface without granulations. Pedipalp fingers with 12-13 rows of granules. Telson with a long aculeus, slightly longer than vesicle, but weakly curved. Dorsal trichobothria of femur, *d*_*4*_ and *d*_*5*_ in a distal position in relation to the external trichobothria *e*_*1*_ .

Description based on female holotype. Morphometric values presented after the description.

Coloration: Ground color is pale yellow to almost whitish; body and pedipalps almost totally pale yellow; legs yellow. Carapace is pale yellow with blackish eyes. Mesosoma yellow with some weak infuscations on tergites I to VI. Metasomal segments I-IV yellow to pale; segment V slightly infuscate ventrally. Vesicle yellow with the aculeus yellow at the base and red at its extremity. Venter is yellow to pale yellow without spots. Chelicerae yellow without any reticulated spots; teeth dark red. Pedipalps yellow to pale yellow overall except for the rows of granules on chela fingers which are red. Legs are yellow.

Morphology. Prosoma: The anterior margin of carapace with a vestigial concavity. Carapace carinae moderately to strongly developed; central median and posterior median carinae moderate to strong; anterior median carinae moderate; central lateral moderate to strong; posterior median and posterior lateral carinae moderate to strong, terminating distally in a small spinoid process that extends very slightly beyond the posterior margin of the carapace. Intercarinal spaces with very few irregular granules, and the reminder of the surface almost smooth, in particular laterally and distally. Median ocular tubercle in a central position and strongly prominent; median eyes large in size and separated by more than two ocular diameters; four pairs of lateral eyes; the fourth largely reduced. Mesosomal tergites I-II pentacarinate; III-VI tricarinate. All carinae strong, granular; each carina terminating distally in a spinoid process that extends slightly beyond the posterior margin of the tergite. Median carinae on I moderate, on II-VI strong, crenulated. Tergite VII pentacarinate, with lateral pairs of carinae strong and fused; median carinae present on the proximal half, moderate to strong. Intercarinal spaces weakly to moderately granular. Lateral carinae absent from sternite III; moderate to strong on sternites IV-VI; strong, crenulate on VII; median carinae on sternites III-IV moderate to strong. Pectines moderately long; pectinal tooth count 28-27. Metasomal segments I-III with ten carinae, moderately crenulate; lateral inframedian carinae on I moderate; on II present on the posterior half; on III limited to a few posterior granules; IV with eight carinae. Dorsal and dorsolateral carinae moderate, without any enlarged denticles distally. All the other carinae moderate to weak on segments I-IV. Segment V with five carinae; ventromedian carinae with several slightly spinoid granules distally; anal arch with three slightly spinoid lobes. Dorsal furrows of all segments weakly developed and smooth; intercarinal spaces almost smooth, with only a few granules on the ventral surface of segment V. Telson almost smooth; subaculear tubercle absent; aculeus slightly longer than vesicle. Chelicerae with two normal denticles at the base of the movable finger [31]. Pedipalps: trichobothrial pattern orthobothriotaxic, type A [29]; dorsal trichobothria of femur in β (beta) configuration [32]. Dorsal trichobothria of femur, *d*_*4*_ and *d*_*5*_ in a distal position in relation to the external trichobothria *e*_*1*_ . Femur pentacarinate; all carinae moderately crenulate. Patella with seven carinae; all carinae moderately to weakly crenulate; dorsointernal carinae with 2-3 spinoid granules. Chelae slender, with elongated fingers; all carinae weakly marked, almost vestigial. Dentate margins of fixed and movable fingers composed of 12-13 almost linear rows of granules. Legs: Ventral aspect of tarsi with short spiniform setae more or less arranged in two rows. Tibial spurs present on legs III and IV, moderately marked. Pedal spurs present on all legs, strongly marked.

**Figure 6.**
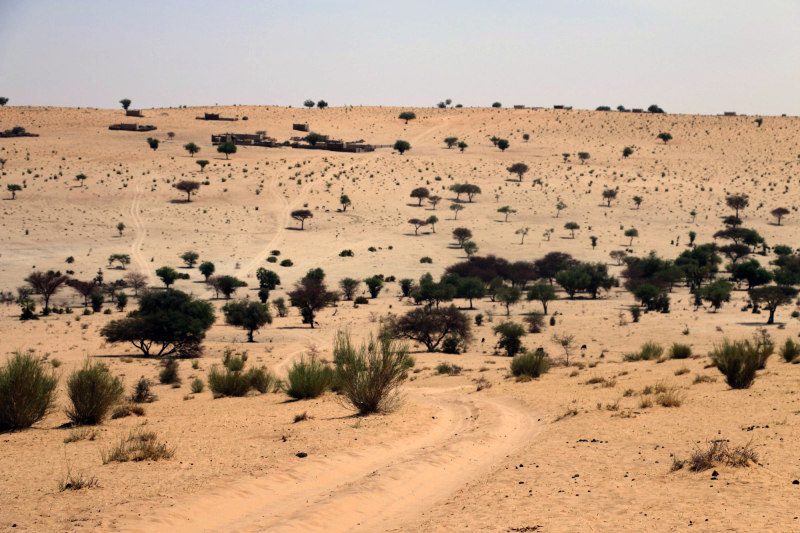
Sahel region in the south of Mauritania, typical habitat of the new species.

Relationships. The new species shows some affinities with *L. hoggarensis* known from the Hoggar region in the south of Algeria. Nevertheless the two species differs by a number of characters: (i) distinct patterns of pigmentation, the population from Hoggar showing a more orange-yellow color while the new species is particularly pale (ii) distinct morphometric values for specimens of a similar global size, (iii) in the new species the telson is less curved with a long aculeus, (iv) dorsal trichobothria of femur, *d*_*4*_ and *d*_*5*_ are disposed in a distal position in relation to the external trichobothria *e*_*1*_ . Moreover, the geographic distributions of the populations are not continuous; in fact *L. hoggarensis* is distributed inside the core region of the desert whereas the distribution of *Leiurus dekeyseri* sp. n. is located in a region of transition between the desert and the Sahel (Figure 6). The future examination of material from Mali should confirm the existence of an intermediate population between those of Algeria and Mauritania.

Morphometric values of the female paratype of *Leiurus hoggarensis* and female holotype of *Leiurus dekeyseri* sp. n. Total length including the telson, 94.6/71.5. Carapace: length 10.5/8.1; anterior width 7.2/5.9; posterior width 12.5/9.7. Mesosoma length: 20.7/15.5. Metasomal segments - I: length 8.2/5.9, width 6.2/4.7; II: length 9.8/6.9, width 5.3/4.0; III: length 10.3/7.4, width 4.9/3.7; IV: length 11.4/8.3, width 4.6/3.4; V: length, 12.5/10.2, width 4.6/3.4, depth 3.9/2.9. Telson length 11.2/9.2; vesicle: width 4.2/3.0, depth 3.8/2.9. Pedipalp: femur length 11.1/8.8, width 2.7/2.2; patella length 12.3/9.7, width 3.2/2.7; chela length 19.9/16.2, width 3.2/2.2, depth 3.3/2.4. Movable finger length 14.4/12.3.

## Conclusions

The main objective of this article is to bring some information about the zoological research on the fields of systematics and taxonomy, which leads to the increasing number of known scorpions of medical importance. This exercise is basically addressed to non-expert people in these domains but who rather use scorpions in their field of research. Attempts are also done to demonstrate that scorpions present complex patterns of diversity and distribution. In account of the group’s diversity is seems important to suggest that the diversity of toxins is most certainly equally complex.

Obviously, the taxonomy and classification of scorpions is far from being a simple task, which causes classification to be permanently changing, a situation most uncomfortable for people using these organisms in their research. Definitely, some toxins are always associated with original species names that have now to be replaced by five to ten redefined new species; the genus *Leiurus* presented here is a perfect example of this situation. Consequently, people employing scorpions in their work should be aware of more precise identification of the species they are using in their research. In a previous publication [3], I have already outlined the numerous situations of possible mixing up that can be found in an important number of scientific publications. In the face of these taxonomic difficulties, the best solution remains to define more important exchanges among professionals using scorpions in their research and true experts. 
